# Incidence of Preeclampsia and Cesarean Section Rate According to the Robson Classification

**DOI:** 10.7759/cureus.49845

**Published:** 2023-12-02

**Authors:** Kritpol Pasokpuckdee, Dittakarn Boriboonhirunsarn

**Affiliations:** 1 Obstetrics and Gynecology, Faculty of Medicine Siriraj Hospital, Mahidol University, Bangkok, THA; 2 Obstetrics and Gynaecology, Faculty of Medicine Siriraj Hospital, Mahidol University, Bangkok, THA

**Keywords:** cesarean rate, cesarean delivery, preeclampsia, cesarean section, robson classification

## Abstract

Objectives

The objective of this study was to determine the incidence of preeclampsia and associated cesarean section (CS) rate according to the Robson classification.

Methods

A retrospective cross-sectional study was conducted on a total of 670 women who delivered at a tertiary care hospital in Thailand during January to March 2023. All women were classified into 10 groups according to the Robson classification, and preeclampsia was identified. Overall and group-specific incidence of preeclampsia and CS rate were estimated. Comparison of CS rate was made between those with and without preeclampsia using the Chi-squared test. Relative risks (RR) and corresponding 95% confidence intervals were estimated.

Results

The majority of women were in group 1 (34%) and group 3 (30.7%). Overall CS rate was 40.6% with highest contribution from group 1, 5, and 10. Incidence of preeclampsia was 9.1%, and the majority were in groups 10 (29.5%) and 1 (23%). Preeclampsia significantly increased the rate of overall CS (RR 1.8, p<0.001). The risk of CS significantly increased in group 1 (RR 1.8, p=0.043), group 3 (RR 3.5, p=0.025), and group 10 (RR 1.9, p=0.006). Preeclampsia accounted for 15.4% of all CS, with the highest contribution in group 2 (37.5%), group 10 (31.1%), group 3 (16.7%), and group 1 (10.8%). Without preeclampsia, the overall CS rate was relatively reduced by 6.9%, with the largest relative reduction in group 10 (14.3%), group 3 (11.5%), group 2 (6.3%), and group 1 (5.2%).

Conclusion

The incidence of preeclampsia was 9.1%, and preeclampsia significantly increased the rate of overall CS. Without preeclampsia, overall CS rate relatively reduced by 6.9% but did not significantly change the relative contribution of CS according to the Robson classification.

## Introduction

Preeclampsia is one of the leading causes of maternal and perinatal mortality worldwide, especially in middle- and low-income countries. It has been estimated that preeclampsia complicates 2-8% of pregnancies globally [1-3]. Preeclampsia was defined as the new onset of hypertension after 20 weeks of gestation, accompanied by significant proteinuria or abnormalities from other laboratory tests or clinical results of organ damage [2, 3]. Hypertensive disorders are responsible for almost 26% of maternal deaths in Latin America and the Caribbean and 9% in Africa and Asia [1-3]. Although preeclampsia is not an indication for cesarean section and vaginal delivery can often be accomplished, an increased rate of cesarean delivery in preeclampsia has been reported [2, 4].

Cesarean section (CS) is the safest route of delivery in certain conditions, such as placenta previa, uterine rupture, etc. [5, 6]. CS without proper indications can increase not only maternal morbidities but also short- and long-term neonatal morbidities [7, 8]. The World Health Organization (WHO) recommends that the rate of CS should be between 10-15% at the population level, but an increasing trend in CS rates has been observed worldwide [6, 9].

The WHO suggests using the Robson classification, which classifies pregnant women into 10 groups based on their obstetric characteristics, for comparison and analysis of CS rates within and across these groups of women, which helps to create strategies to optimize CS rates [6]. Robson classification can be used as a standard tool for assessing, monitoring, and comparing cesarean rates among facilities, countries, and regions. It can also be used to develop guidelines for reducing the rate of CS [6, 10].

In Thailand, recent reports from two university hospitals showed that the overall CS rates were as high as 48.9% and 55.5% [11, 12]. Nonetheless, there are only a few previous studies that related the Robson classification to women with some obstetric characteristics or specific conditions [4, 13, 14]. A previous study reported that preeclampsia was most common among women in group 10 of the Robson classification, and the CS rate was significantly increased among women with preeclampsia, especially among women in groups 1, 5, and 10 [4]. However, in Thailand, there is no study that evaluates the contribution of obstetric complications, such as preeclampsia, to the CS rate in each group of the Robson classification.

Therefore, the objectives of this study were to determine the incidence of preeclampsia according to the Robson classification and to evaluate the contribution of preeclampsia to the CS rate in each group of the Robson classification. 

## Materials and methods

After approval from the Siriraj Institutional Review Board (SIRB), a cross-sectional study was conducted at the Department of Obstetrics and Gynecology, Faculty of Medicine, Siriraj Hospital, which is the largest university-based tertiary care hospital in Thailand. All pregnant women who delivered at Siriraj Hospital from January to March 2023 were included. The sample size was calculated from an estimated prevalence of preeclampsia of 15%. At a 95% confidence level and 3% acceptable error, at least 545 cases were needed.

All women who delivered during the study period were included and then classified into 10 groups according to the Robson classification (Table 1). Preeclampsia was diagnosed when there was a new onset of hypertension after 20 weeks of gestation (systolic blood pressure of >140 mmHg or diastolic blood pressure of >90 mmHg on two occasions at least four hours apart), accompanied by significant proteinuria (>300 mg from 24-hour urine collection or protein/creatinine ratio of >0.3 mg/dL or dipstick reading of >2+, or abnormalities from other laboratory tests or clinical results of organ damage (e.g., thrombocytopenia, renal insufficiency, impaired liver function) [2, 3].

**Table 1:** Robson classification Robson classification from the WHO Statement on Cesarean Section Rates [6]

Robson classification	Characteristics
Group 1	Nulliparous with single cephalic pregnancy, ≥37 weeks gestation in spontaneous labor
Group 2	Nulliparous with single cephalic pregnancy, ≥37 weeks gestation who either had labor induced (2a) or were delivered by cesarean section before labor (2b)
Group 3	Multiparous without a previous uterine scar, with single cephalic pregnancy, ≥37 weeks gestation in spontaneous labor
Group 4	Multiparous without a previous uterine scar, with single cephalic pregnancy, ≥37 weeks gestation who either had labor induced (4a) or were delivered by cesarean section before labor (4b)
Group 5	All multiparous with at least one previous uterine scar, with single cephalic pregnancy, ≥37 weeks gestation
Group 6	All nulliparous women with a single breech pregnancy
Group 7	All multiparous women with single breech pregnancy, including women with previous uterine scars
Group 8	All women with multiple pregnancies, including women with previous uterine scars
Group 9	All women with a single pregnancy with a transverse or oblique lie, including women with previous uterine scars
Group 10	All women with a single cephalic pregnancy <37 weeks gestation, including women with previous scars

Antepartum and intrapartum management of preeclampsia was done according to institutional guidelines by attending residents or fellows under staff supervision. This includes the use of magnesium sulfate for the prevention of preeclampsia, antihypertensive drugs, and other symptomatic and supportive treatments, as indicated. The decision to perform cesarean delivery was individualized, based on the anticipated probability of vaginal delivery and on the nature and progression of preeclampsia, as well as other obstetric indications, at the discretion of the clinical care team.

Data were extracted from medical records, including baseline characteristics, obstetric data, mode of delivery, and pregnancy outcomes. The diagnosis of preeclampsia was reviewed and verified. Overall and group-specific CS rates were determined and compared between those with and without preeclampsia according to the Robson classification. The overall and group-specific relative contribution of CS in preeclampsia was determined. Changes in CS rate after excluding preeclampsia cases were evaluated in terms of absolute change in percentage points and relative change in percent.

Descriptive statistics, including mean, standard deviation, number, and percentage, were used to describe various characteristics as appropriate. The Chi-squared test or Fisher's exact test were used to compare various characteristics between those with and without preeclampsia. Relative risk (RR) and 95% confidence intervals (CI) were estimated to determine the association between preeclampsia and CS rate. A p-value of < 0.05 was considered statistically significant. All analyses were performed using SPSS version 21.0 (IBM Inc., Armonk, New York).

## Results

A total of 670 pregnant women were included in this study. Table 2 shows the baseline characteristics of the women. The mean age was 29.7 years, and the mean BMI was 24.0 kg/m^2^; 313 women (46.7%) were nulliparous, and 234 women (33.4%) were overweight or obese. Gestational diabetes was found in 13.7% (92 women), and preeclampsia occurred in 61 women (9.1%, 95%CI 7.0-11.5). The gestational age at delivery was 37.7 weeks of gestation, and the mean birth weight was 2945 g. Small for gestational age infants was found in 8.9%, and there was no fetal death.

**Table 2:** Baseline characteristics of the study population (N=670) GA - gestational age

Characteristics	N (%)
Mean age ± SD (years)	29.7 ± 6.2
Mean BMI ± SD (kg/m^2^)	24.0 ± 4.5
Nulliparity	313 (46.7)
BMI category	
Underweight	43 (6.4)
Normal	403 (60.1)
Overweight	169 (23.7)
Obesity	65 (9.7)
Gestational diabetes	92 (13.7)
Chronic hypertension	20 (3.0)
Preeclampsia	61 (9.1)
Mean GA at delivery ± SD (weeks)	37.7 ± 2.5
Mean birth weight ± SD (g)	2945.0 ± 597.3
Small for gestational age	60 (8.9)

Table 3 demonstrates the Robson classification of all the women. The majority of women were in group 1 (228 women, 34%), group 3 (206 women, 30.7%), group 10 (92 women, 13.7%), and group 5 (64 women, 9.6%). The overall CS rate was 40.6% (272 of 670). Groups 1, 5, and 10 had the highest contribution to CS of 27.2% (74 women), 23.2% 63 women), and 16.5% (45 women), respectively. As there is no policy on trial of labor after CS unless delivery is imminent, women in group 5 had a 98.4% CS rate (63 of 64 women). CS rate was 32.5% in group 1 (74 of 228 women), and it was as high as 48.9% in group 10 (45 of 92 women).

**Table 3:** CS rate according to the Robson classification (N=670) CS - cesarean section

Group	Women in group N (%)	CS rate in group N (%)	Relative contribution of CS (%)
1	228 (34.0)	74 (32.5)	27.2
2	16 (2.7)	16 (88.9)	5.9
3	206 (30.7)	18 (8.7)	6.6
4	8 (1.2)	5 (62.5)	1.8
5	64 (9.6)	63 (98.4)	23.2
6	14 (2.1)	14 (100)	5.1
7	12 (1.8)	12 (100)	4.4
8	24 (3.6)	21 (87.5)	7.7
9	1 (0.6)	4 (100)	0.6
10	92 (13.7)	45 (48.9)	16.5
Total	670 (100)	272 (40.6)	100

Overall, 61 women had preeclampsia, corresponding to an incidence of 9.1% (95%CI 7.0-11.5), and the distribution in each group of the Robson classification is shown in Table 4. Majority of preeclampsia cases were in group 10 (18 cases, 29.5%), group 1 (14 cases, 23%), and group 3 (11 cases, 18%). The distribution of women without preeclampsia was similar to that of overall cases. In terms of severity, 29 of 61 cases (47.5%) were preeclampsia with severe features. A higher CS rate was observed in preeclampsia with severe features than those without (23 of 29; 79.3% vs. 20 of 33; 59.4%) without statistical significance (p=0.093). Further subgroup stratification by its severity into the Robson classification showed too few cases in each group that resulted in less reliable estimates of CS rates.

**Table 4:** Distribution of preeclampsia among the Robson classification group (N=670)

Group	Preeclampsia N=61	No preeclampsia N=609
1	14 (23%)	214 (35.1%)
2	6 (9.8%)	12 (2.0%)
3	11 (18%)	195 (32.0%)
4	0 (0%)	8 (1.3%)
5	0 (0%)	64 (10.5%)
6	4 (6.6%)	10 (1.6%)
7	2 (3.3%)	10 (1.6%)
8	5 (8.2%)	19 (3.1%)
9	1 (1.6%)	3 (0.5%)
10	18 (29.5%)	74 (12.2%)
Total	61 (100%)	609 (100%)

Table 5 demonstrates the CS rate in each group of the Robson classification, according to preeclampsia diagnosis. Overall, preeclampsia significantly increased the rate of CS compared to those without (68.9% vs. 37.8%; RR 1.8, 95%CI 1.5-2.2; p<0.001). In addition, the risk of CS also significantly increased among women with preeclampsia in group 1 (57.1% vs. 30.8%; RR 1.8, 95%CI 1.1-3.0; p=0.043), group 3 (27.3% vs. 7.7%; RR 3.5, 95%CI 1.2-10.5; p=0.025), and group 10 (77.8% vs. 41.9%; RR 1.9, 95%CI 1.3-2.7; p=0.006).

**Table 5:** CS rate of women with and without preeclampsia according to Robson classification (N=670) PE - preeclampsia; CS - cesarean section; RR - relative risk

Group	All cases N (%)	PE N (%)	No PE N (%)	RR (95%CI)	p-value
1	74 (32.5)	8/14 (57.1)	66/214 (30.8)	1.8 (1.1-3.0)	0.043
2	16 (88.9)	6/6 (100)	10/12 (83.3)	1.2 (0.9-1.5)	0.290
3	18 (8.7)	3/11 (27.3)	15/195 (7.7)	3.5 (1.2-10.5)	0.025
4	5 (62.5)	0 (0)	5/8 (62.5)	NA	NA
5	63 (98.4)	0 (0)	63/64 (98.4)	NA	NA
6	14 (100)	4/4 (100)	10/10 (100)	NA	NA
7	12 (100)	2/2 (100)	10/10 (100)	NA	NA
8	21 (87.5)	5/5 (100)	16/19 (84.2)	1.2 (0.9-1.4)	0.342
9	4 (100)	1/1 (100)	3/3 (100)	NA	NA
10	45 (48.9)	14/18 (77.8)	31/74 (41.9)	1.9 (1.3-2.7)	0.006
Total	272 (40.6)	42/61 (68.9)	230/609 (37.8)	1.8 (1.5-2.2)	<0.001

Preeclampsia accounted for 15.4% of all CS cases (42 of 230 cases). According to the Robson classification, the contribution of preeclampsia to CS was highest in group 2 (6 of 16 cases, 37.5%), followed by group 10 (14 of 45 cases, 31.1%), group 3 (3 of 18 cases, 16.7%), and group 1 (8 of 74 cases, 10.8%). When women with preeclampsia were excluded, the overall CS rate decreased from 40.6% (272 of 670) to 37.8% (230 of 609), with a relative reduction of 6.9%. For each Robson classification group, the largest relative reduction in CS rate was in group 10 (14.3%), followed by group 3 (11.5%), group 2 (6.3%), and group 1 (5.2%).

Further analysis showed that if preeclampsia cases were not included, the distribution of the relative contribution of CS in each Robson classification group did not significantly change from those of all cases. Group 1 showed the highest contribution of 28.9% (66 of 230 cases), followed by group 5 (63 of 230 cases, 27.4%), and group 10 (31 of 230 cases, 13.5%).

## Discussion

The Robson classification is recommended for use in evaluating and monitoring the CS rate. However, the classification also includes women with obstetric complications, such as preeclampsia. Such inclusion could probably interfere with the CS rate and interpretations of the results, especially for specific conditions with a high CS rate.

The incidence of preeclampsia in this study was 9.1%, and of them, 29.5% were diagnosed before 37 weeks of gestation (group 10). This was slightly higher than the estimated incidence of 2-8% of pregnancies globally [1, 2]. This could be because the study hospital is a tertiary care referral center. According to the Robson classification, 29.5% of preeclampsia were in group 10, i.e., diagnosed before 37 weeks of gestation. The results also showed that 46.7% of cases were nulliparous, as nulliparity is one of the important risks for preeclampsia [2].

The results demonstrated that the overall CS rate was almost doubled among those with preeclampsia, i.e., RR 1.8, 95%CI 1.5-2.2, and a significant increase in CS rate was also observed in groups 1, 3, and 10 (RR between 1.8 and 3.5). Similar results were also observed from a previous study, which found that preeclampsia significantly increased the risk of CS by 2.2 times, especially in groups 1 and 10 [4].

Although preeclampsia is not an absolute indication for CS and vaginal delivery can often be accomplished, the rate of CS is usually high, partly due to the severity of the disease, which can result in severe maternal complications, and fetal compromises and immediate delivery is commonly recommended [1-3]. The risk of CS was also reported to increase with decreasing gestational age of diagnosis, and a CS rate of as high as 77.8% among women in group 10 was observed in this study [2].

If women with preeclampsia were excluded, the overall and group-specific CS rate would decrease, with the largest relative reduction in group 10 (14.3%). Although preeclampsia accounted for 15.4% of all CS cases, further analysis revealed that the relative contribution of CS in each group of the Robson classification did not change significantly after the exclusion of preeclampsia cases.

The results showed that the overall CS rate was 40.6%. The rate was slightly lower than the previous report from the same institution of 48.9% and 55.5% from the other tertiary care university hospitals [11, 12]. This might be from various efforts over the past few years to reduce unnecessary CS. Both clinical and non-clinical interventions have been implemented as recommended by various authorities, including continuous monitoring of the CS rate by Robson classification, improvement of the intrapartum care process, a mandatory second opinion, and an audit and feedback system [5, 15].

Robson groups 1-4 (term, nulli- or multiparous, ≥37 weeks of gestation, with cephalic presentation) are considered at lower risk for CS, with an expected rate of 10.0%, 35.0%, 3.0%, and 15.0%, respectively [16]. However, all the CS rates exceeded these expectations, which might be due to many factors, including information the women received during antenatal care, cultural beliefs and norms, variations in intrapartum decision-making, and fear of medio-legal claims, which varied in different contexts of care. Group 1 had the highest contribution of 27.2%, which was similar to what was observed from previous studies in Thailand as well [11, 12]. The high CS rate in group 1 of 32.5% of the women and as high as 30.8% in those without preeclampsia will need to be explored more in detail, and further specific interventions should be designed to reduce such a high rate. The actual reasons behind the high CS rates should be examined further. An audit and feedback system could also help identify some of the problems in clinical care that could lead to more appropriate clinical interventions or strategies to reduce the CS rate without increasing morbidities and risk of medico-legal claims in the future.

The strength of this study is that it is the first study in Thailand to evaluate the contribution and impact of preeclampsia on the CS rate according to the Robson classification. 

The limitations of this study included the retrospective design, which might make some information less accurate. However, all the information was carefully reviewed, and preeclampsia diagnosis was verified in every case. Misclassification of the Robson classification could occur but should be less likely since the data were routinely collected in a prospective manner after each delivery, and all the variables to classify the women were objectively evaluated. The actual reasons behind the decision for CS could not be accurately determined. The relatively small number of preeclampsia in each group could lead to less accurate results. Selection bias was unlikely since all the women were included, and the characteristics and distribution of women in the Robson classification were similar to a previous report from the same hospital [11]. Further studies with prospective data collection could minimize possible inaccuracy and biases. A larger study with larger samples of preeclampsia cases could also provide more information on the correlation with CS rate in each group of the Robson classification.

## Conclusions

In conclusion, the CS rate was significantly higher in preeclampsia but did not significantly change the relative contribution of CS according to the Robson classification. This provides clinicians and policy-makers a better understanding of the CS rate and can be used as baseline information to accurately interpret the Robson classification in the future. Further prospective studies are still needed, with larger samples to explore in detail how preeclampsia and other serious complications impact the CS rate in each specific facility.
